# Organization of nursing care in three Nordic countries: relationships between nurses’ workload, level of involvement in direct patient care, job satisfaction, and intention to leave

**DOI:** 10.1186/1472-6955-13-27

**Published:** 2014-10-06

**Authors:** Rikard Lindqvist, Lisa Smeds Alenius, Sara Runesdotter, Anneli Ensio, Virpi Jylhä, Juha Kinnunen, Ingeborg Strømseng Sjetne, Christine Tvedt, Maria Wiberg Tjønnfjord, Carol Tishelman

**Affiliations:** 1 Medical Management Centre, Department of Learning, Informatics, Management and Ethics, Karolinska Institutet, 171 77 Stockholm, Sweden; 2 Department of Health and Social Management, University of Eastern Finland, Kuopio, Finland; 3 Norwegian Knowledge Centre for the Health Services, Oslo, Norway; 4 Institute of Health and Society, University of Oslo, Oslo, Norway; 5 Lovisenberg Diaconal University Collage, Oslo, Norway; 6 Department of Health, Nutrition and Management, Oslo and Akershus University College of applied sciences, Faculty of Health Sciences, Akershus, Norway

**Keywords:** Health services research, Job satisfaction, Nursing, Organization and administration, Workload

## Abstract

**Background:**

Health care systems in Finland, Norway and Sweden share many similarities, e.g. full-coverage and tax-financed, with predominately public sector hospitals. Despite similarities, there are differences in the working situations for RNs within these Nordic countries. The aim of this study was to analyze associations between RNs’ patient workload and level of involvement in direct patient care, their job satisfaction and intention to leave in these countries.

**Methods:**

A workforce survey was conducted through RN4CAST, an EU 7th framework project. The survey included 118 items derived from validated instruments or tested in prior research. Responses from 1133 RNs at 32 Finnish hospitals, 3752 RNs at 35 Norwegian hospitals, and 11 015 RNs at 71 Swedish hospitals comprise the database, which was analyzed using logistic and odds ratio regressions analyses.

**Results:**

We found statistically significant differences in RNs’ level of involvement in direct patient care (p < 0.001, Sweden compared to Norway and Finland), in patient workload and in number of patients needing ADL assistance and surveillance. A U-formed relationship was found between level of involvement in direct patient care and intention to leave in Sweden, and more satisfaction among RNs in roles with more direct patient care (OR = 1.16, 1.02 ≤ CI_95%_ ≤ 1.32). Nearly half the Finnish sample report intention to leave, with significantly lower levels in Norway and Sweden (p < 0.001). Patient workload is associated with job satisfaction and intention to leave to some degree in all countries, i.e. greater patient workload, less job satisfaction and greater intention to leave.

**Conclusions:**

This study suggests that more attention paid to patient mix, workload and role of RNs in patient care might potentially diminish intention to leave and increase job satisfaction in these Nordic countries.

## Introduction and aim

The health care systems (HCS) in the Nordic countries share many features. Finland, Norway and Sweden all share principles of equal access to care, with health care primarily provided by the public sector and thus subject to government control. The HCSs in all three countries are largely full-coverage and tax-financed. In addition, these three countries have all undergone public reforms and changes in their respective HCS since the early 1990s to increase patient influence and economic efficiency [1,2].

Despite these similarities in HCSs, a comparative study based on European data from the EU (European union) 7^th^ framework RN4CAST project (Nurse Forecasting: Human Resources Planning in Nursing 2009 – 2011) [3,4] indicates substantial differences in the working conditions of RNs (registered nurse) in these three Nordic countries. Aiken et al. [4] found for example that self-reports from RNs in Norway indicated one of the lowest proportions of intention to leave their current hospital - (25%) among the 12 participating countries, while, the RNs in Finland reported one of the highest proportions (49%), with Swedish RNs between (34%) these Nordic extremes. However the proportion of RNs reporting that they would *not* recommend the hospital as workplace to other RNs was 26% in Sweden compared to 17% in Finland and 14% in Norway [4]. In addition, comparative economic studies of Nordic health care systems have determined that Finnish hospitals are most cost efficient, but these studies have not considered RN-related variables ([5]). The combination of common features and clear differences among the Nordic countries is intriguing, and access to this unique material stimulated this investigation of how differences in the organization of nursing work are related to central outcome variables like RN job satisfaction and intention to leave the workplace or profession.

**The aim of this study** is therefore to explore associations between RNs’ patient workload and level of involvement in direct patient care, and RNs’ job satisfaction and intention to leave their current hospital or the nursing profession in Finland, Norway and Sweden.

Specific research questions addressed include:

•Does RNs’ level of involvement in direct patient care, patient workload, job satisfaction, and intention to leave differ among the three Nordic countries?

•Is RNs’ patient workload associated with job satisfaction and intention to leave?

•Is RNs’ level of involvement in direct patient care associated with job satisfaction and intention to leave?

•To what extent does workload interact with the RNs’ level of involvement in direct patient care with regard to job satisfaction and intention to leave?

Investigating these issues among countries with such similar HCSs will both provide new knowledge of interest for policy decisions, and will serve to generate new hypotheses for further investigation in countries with different HCSs.

## Background

### Health care organization in Finland, Norway and Sweden

In these three Nordic countries, in-hospital health care provision is the responsibility of regional authorities with hospitals providing specialized services for their catchment area; Finland has 20 hospital districts, Sweden has 21 health care authorities, and Norway has four. While specific health care financing mechanisms between different levels of government and individual hospitals vary, the out-of-pocket costs for hospital care for individuals in all three countries are <5% of the total cost [6]. Another commonality is that the overwhelming majority of acute care hospitals are owned and operated by the public sector, with patients generally referred to hospital via primary care, except in case of emergency.

Three categories of nursing staff can be found in these Nordic countries, although in acute care settings, all have shifted to a predominance of RNs during the past few decades. RN education also is relatively comparable among the countries. All have undergone a shift from technical and non-academic to academically-based nursing education, which means that the RN workforce has an educational mix, resulting in an inverse relationship for many between length of experience and educational level. At present, RN education is provided at universities or university colleges, although in Finland it is a half year longer (210 ECTS - European Credit Transfer System points) than the three-year programs in Norway (180 ECTS) and Sweden (180 ECTS). Since the early part of this century these programs all lead to both a Bachelor Degree and RN licensure [7]. Assistant or practical nurses have upper secondary school level vocational education. The programs for Nurse’s Aides or Assistants are shorter, although this category of nursing staff has radically decreased in acute care hospital services in all three countries in recent years.

Between 80-90% of clinically-active RNs are unionized in Finland, Norway and Sweden ([8], (Norsk Sykepleierforbund [Norwegian Nurses Organisation], personal correspondence Aug 1, 2011), (Vårdförbundet [Swedish Association of Health Professionals] Per Malmquist, personal correspondence Feb 1, 2011)). This may be related to historical factors, e.g. traditions of collective bargaining between unions and employer organizations, and in Finland and Sweden, unemployment insurance originally based on union affiliation. All three countries also have strong national regulation of working times and vacation norms, although these vary among countries. In Finland and Sweden, a fulltime (FT) position for an RN working shifts is 38.25 hours/week, while Norway defines FT as 35.5 hours. All three countries have a minimum of 4–5 weeks paid vacation.

## Material and methods

### Study design

The data analyzed here derive from the EU 7^th^ framework project, RN4CAST [3]. Nurses have been surveyed as informants about organizational characteristics (e.g. nursing work environment) at the nursing unit and hospital level as well as about individual nurse outcomes, e.g. job satisfaction and both intention to leave the hospital and intention to leave the nursing profession. Data was collected from RNs working in general acute care hospitals with direct patient care for adults or mixed age groups. While national recruitment could vary somewhat between countries for pragmatic reasons, some features were common to all RN4CAST countries. These include an effort to select hospitals in both urban and rural regions, including both teaching and non-teaching facilities. Acute-care medical and surgical units were included, with the exception of various types of intensive care and high dependency units. Pediatric, chronic care and long-term nursing care units (e.g. geriatric units) were not included.

## Methods

### Data sources/measurement

The RN4CAST nurse survey consisted of a core battery of 118 items derived from well-known and validated instruments, as well as questions developed and tested in prior research; all were translated for this purpose ([9,10] see also [3,11] for further information). Translations were validated using a content validity index [12] (0.79 for the Finnish version, 0.72 for the Norwegian, and 0.91 for the Swedish version).

To address the research questions posed here, nine items from the RN4CAST nurse survey questionnaire were analyzed. The items were categorized into four basic constructs: job satisfaction (2 items), *workload* (4 items), *level of involvement in direct patient care* (1 item) and *intention to leave* (2 items). Table 1 presents these items with their response alternatives.

**Table 1:** Constructs, questions and response alternatives used in the study

**Construct**	**Question**	**Response scale**
*Job satisfaction*		
	How satisfied are you with your current job in this hospital?	1 = Very dissatisfied
		2 = A little dissatisfied
		3 = Moderately satisfied
		4 = Very satisfied
	Would you recommend your hospital to a nurse colleague as a good place to work?	1 = Definitely no
		2 = Probably no
		3 = Probably yes
		4 = Definitely yes
*Workload*		
	How many patients were you directly responsible for on the most recent shift you worked?	Count
	Is the number of patients in preceding question typical of your workload?	1 = Less
		2 = Same
		3 = More
	Of all the patients you were directly responsible for on your most recent shift, ..	
	How many required assistance with all activities of daily living?	Count
	How many required hourly or more frequent monitoring or treatments?	Count
*Level of involvement in direct patient care*		
	How would you describe your role in caring for most of the patients on your most recent shift?	1 = I provided most care myself
		2 = I supervised the care by others and provided some myself
		3 = I provided only limited care such as dressing changes or drug administration and most of direct care was done by others
*Intention to leave*		
	If possible, would you leave your current hospital within the next year as a result of job dissatisfaction?	Yes/No.
	*Follow up question:* If yes, what type of work would you seek?	1 = Nursing in another hospital
		2 = Nursing, but not in a hospital
		3 = Non-nursing

### Participants

#### Finland

Purposive, stratified sampling based on geographic location was used to select hospitals with >100 patient beds which provided acute care. This approach allowed recruitment of facilities in different hospital districts, in urban and rural areas including all the major cities, and different types of hospitals (university, central hospitals, and city hospitals), with at least one university or central hospital selected from each district. The Helsinki-Uusimaa district, comprised of five hospitals and providing health care for 28% of the population, was an exception; recruitment was adapted here to allow for representativity in this area. Thirty-two of the 34 hospitals invited to participate in the study agreed; survey data was then collected from a minimum of two general medical/surgical units per hospital.

The RN survey was conducted in Finland from November 2009-February 2010. A total of 2,463 RNs at 130 units were asked to participate by an email invitation sent to each RN at either a personal email address or to a hospital unit email, by either the researchers or the head nurse of their unit, depending on hospital policy. The email invitation contained a link to the web-based questionnaire. A first reminder was sent after one week, with most hospital units sent two reminders. Hospitals with RN response rates <40% were contacted again in February 2010, and asked to make further efforts to motivate RNs to respond to the survey. The final response rate per hospital varied from 27-62%.

#### Norway

As national registration of up-to-date RN workplace information does not exist, the survey was distributed via the Norwegian Nurses’ Organization’s (NNO) local representatives at acute care hospitals with a minimum of 90 patient beds (36 hospitals). They received 6,600 paper questionnaires with survey ID numbers distinguished by workplace, and distributed them to RN colleagues. Completed questionnaires were returned to the Norwegian Knowledge Centre for the Health Services. This procedure did not allow for systematic reminders to be distributed; instead the local NNO-representatives were asked to encourage and remind RNs to respond.

The sample included all but one of the hospitals large enough for survey inclusion (35 hospitals). The response rate per hospital varied from 24 to 86%.

**Sweden** also collaborated with the union organizing RNs, the Swedish Association of Health Professionals, using their member register for recruitment to the RN survey. As the register contains self-reported workplace information on both hospital and department, but without information as to specific nursing function, all RNs registered as working in medical or surgical departments were included. Residential addresses were linked to this sample via Sweden’s system of individually unique national registration numbers. The survey was then posted in February 2010 to over 33,000 RNs’ residential addresses by Statistics Sweden, with the option of either returning it by pre-paid mail or completing a web-based version. Three reminders were sent at standard intervals, with the last two each containing a new copy of the survey. The return rate was just under 70%, with hospital response rates varying from 53- 81%.

Final study inclusion was based on responses to the first question, designed to eliminate conscious over-recruitment, i.e. RNs not meeting study criteria by for example, not working actively with in-patient care, working in non-medical/surgical units, or in ICUs or operating rooms. The second question was to verify or change workplace data derived from the register; respondents who had changed workplace or did not otherwise meet inclusion criteria were also excluded.

The recruitment strategies used in Sweden allowed a non-respondent bias analysis based on known background variables (age, gender and workplace) to be performed, with no systematic differences detected between responders and non-responders. Since the material included a known over-recruitment, analysis of background variables was also conducted to establish if there were systematic differences in response rates between the study group and the over-recruitment group, without any systematic difference detected.

### Data analysis

Mean averages and confidence intervals for ratio scale items (*age, experience, number of patients cared for on last shift*) for the samples in each country were calculated. Country differences in the ordinal scale item, *job satisfaction*, were analyzed using Mann–Whitney *U*-test. Differences in proportions to verify differences between countries for other descriptive variables were analyzed using Chi^2^-tests. Proportion odds models were used to determine if *involvement in direct patient care* and *workload* were associated with *job satisfaction*, controlling for *age, sex, length of work experience, hospital* and *reported number of nursing staff*. In regression analyses, job satisfaction was operationalized using the item *How satisfied are you with your current job in this hospital?* as this item showed most variability. Logistic regression analyses were used to determine if *involvement in direct patient care* and *workload* were associated with the dichotomous outcome variable *intention to leave current hospital* (yes/no), controlling for *age, sex, length of work experience, hospital* and *reported number of nursing staff*. By controlling for *hospital*, we aimed to eliminate the effects of variability due to differences in work environments. (see Table 1 for response alternatives).

A stepwise approach was used for both series of regression analyses, beginning with results of bivariate analyses and adding variables thereafter, with one model fit for each country. Score Test and plots of the logits were used to control the Proportional Odds Assumption. The level of statistical significance (α) employed was 0.05 for all analyses.

### Ethical considerations

The project was approved by the relevant Research Ethics Committee in respective country (In Finland: Central Ethics Committee of the Northern Savo hospital district, in Norway Data Protection Official for research and in Sweden: Central Ethical Review Board in Stockholm). All participants were informed that the collected data would be analyzed in anonymous form and that participation was voluntary. Completing and returning the survey was considered a sign of informed consent by the ethical committees.

## Results

The vast majority of the respondents in all countries were women; 6% were men in Norway and Sweden with the proportion in Finland significantly lower (3%). The Finnish sample was both statistically significantly older (mean age 41.7) and reported longer nursing experience (mean 14.1 years’ experience) than the participating RNs in Sweden (mean age 40.2; 12.1 years’ experience) and Norway (mean age 35.5; 8.5 years’ experience).

More respondents in Norway (5%) than in the other countries (2.3% in Sweden; <1% in Finland) reported having received their basic nursing education outside the country, with 27% of these Norwegian respondents educated in Sweden and 7% in Finland. Twenty-seven percent of the RN respondents from the Swedish sample who reported a foreign nursing education were educated in Finland and 5% in Norway (data not shown). Approximately 1% of the Finnish and Norwegian RNs reported having worked in another Nordic country; 0.4% of the Swedish sample reported having had worked in Finland, whereas 9% had worked in Norway (data not shown).

### RNs’ level of involvement in direct patient care

There are distinct statistically significant differences (p < 0.001) regarding the reports of the RN level of involvement in direct patient care between Sweden on one hand, and Norway and Finland on the other (see Table 2). Approximately 27% of RNs in Sweden reported providing most care his/herself, whereas the corresponding proportions were 58% in Finland and 65% in Norway. The majority of Swedish nurses (52%) reported supervising care by others and providing some care his/herself, whereas this was the case for approximately one quarter of RN respondents from both Finland and Norway.

**Table 2:** Descriptive statistics of outcomes and independent variables

	**Finland**		**Norway**		**Sweden**		**P-value**		
	**N**	**Per-cent**	**N**	**Per-cent**	**N**	**Per-cent**	**FI vs NO**	**FI vs SW**	**NO vs SW**
**Level of involvement in direct patient care**									
*How would you describe your role in caring for most of the patients on your most recent shift? Mark the one option that fits best.*									
I provided most care myself	657	58%	2,448	65%	3,016	27%	0.0641^a^	<0.0001^a^	<0.0001^a^
I supervised the care by others and provided some myself.	302	27%	940	25%	5,697	52%			
I provided only limited care such as dressing changes or drug administration and most of direct care was done by others	89	8%	291	8%	1,806	16%			
Missing	85	8%	73	2%	496	5%			
*Total*	*1,133*	*100%*	*3,752*	*100%*	*11,015*	*100%*			
**Patient workload**									
*How many patients were you directly responsible for on the most recent shift you worked?*									
Overall mean (Low ≤ CI 95% ≤ High)		9.42 (9.06-9.78)		6.54 (6.42-6.66)		7.59 (7.50-7.68)	<0.0001	<0.0001	<0.0001
*Is the number of patients in preceding question typical of your workload?*									
Less	439	39%	1,252	33%	769	7%	<0.0001^a^	<0.0001^a^	<0.0001^a^
Same	497	44%	2,090	56%	6,790	62%			
More	128	11%	332	9%	2,729	25%			
Missing	69	6%	78	2%	727	7%			
*Of all the patients were you directly responsible for on your most recent shift, how many required assistance with all activities of daily living?*									
Overall mean (Low ≤ CI 95% ≤ High)		4.47 (4.24-4.70)		3.04 (2.97-3.12)		2.71 (2.66-2.76)	<0.0001	<0.0001	<0.0001
*Of all the patients were you directly responsible for on your most recent shift, how many required hourly or more frequent monitoring or treatments?*									
Overall mean (Low ≤ CI 95% ≤ High)		3.55 (3.36-3.74)		1.92 (1.84-2.00)		2.64 (2.58-2.70)	<0.0001	<0.0001	<0.0001
**Job satisfaction and intention to leave**									
*How satisfied are you with your current job in this hospital?*									
Very dissatisfied	38	3%	94	3%	468	4%	<0.0001^b^	<0.0001^b^	<0.0001^b^
A little dissatisfied	262	23%	679	18%	1,937	18%			
Moderately satisfied	665	59%	2,071	55%	6,161	56%			
Very satisfied	149	13%	885	24%	2,284	21%			
Missing	19	2%	23	1%	165	1%			
*Would you recommend your hospital to a nurse colleague as a good place to work?*									
Definitely no	12	1%	59	2%	433	4%	<0.0001^b^	<0.0001^b^	<0.0001^b^
Probably no	171	15%	455	12%	2,353	21%			
Probably yes	693	61%	2,183	58%	5,758	52%			
Definitely yes	229	20%	1,034	28%	2,223	20%			
Missing	28	2%	21	1%	248	2%			
*If possible, would you leave your current hospital within the next year as a result of job dissatisfaction?*									
Yes	546	48%	942	25%	3,651	33%	<0.0001^c^	<0.0001^c^	<0.0001^c^
No	565	50%	2,770	74%	7,181	65%			
Missing	22	2%	40	1%	183	2%			
*If yes, what type of work would you seek?*									
Nursing in another hospital	277	51%	344	30%	1,134	31%	<0.0001^a^	<0.0001^a^	<0.0001^a^
Nursing, but not in a hospital	154	28%	417	37%	1,470	40%			
Non-nursing	114	21%	375	33%	765	21%			
Missing	1	0.2%			282	8%			

^a^Chi-square computation was based only on valid cases; df =2.

^b^Mann–Whitney U computation was based only on valid cases.

^c^Chi-square computation was based only on valid cases; df =1.

### Patient workload

The mean number of patients the RNs reported being directly responsible for on the most recent shift differed significantly (p < 0.001) among all three countries, with Finnish nurses reporting about 40% more patients (9.4) compared to Norway (6.5) and Sweden (7.6) (see Table 2).

As shown in Table 2, RNs in Finland reported the highest mean number of patients requiring assistance with activities of daily living (ADL) on their most recent shift (4.5), followed by those in Norway (3.0) and Sweden (2.7), with these differences statistically significant (p < 0.001) among all countries. The Finnish RNs also reported the highest number of patients requiring frequent monitoring or treatments (3.6).

### Job satisfaction

The distribution of responses to the question on job satisfaction differed significantly (p < 0.001) among countries (see Table 2). When responses were dichotomized, the highest proportion of RNs reporting being moderately or very satisfied with her/his current job was found in Norway (79%), with comparable reports from 77% of the Swedish sample and 72% of the Finnish with differences between Finland and both the other Nordic countries, statistically significant (data not shown). The proportion of RNs reporting being very dissatisfied was 3-4% in all three countries, whereas the proportion of those reporting being a little dissatisfied was significantly (p < 0.001) higher in Finland (23%) than in Sweden and Norway (both 18%).

Eighty-six percent of the respondents from Norway reported that they would probably or definitely recommend their hospital to a RN colleague as a good place to work, with 81% of the Finnish sample responding similarly. Responses from both Finland and Norway differed significantly (p < 0.001) from the Swedish sample, in which 72% would recommend her/his hospital as a good workplace (see Table 2).

### Intention to leave

About half (48%) of the RN respondents from Finland reported that, if possible, they would leave their current hospital within the next year due to job dissatisfaction, with approximately one-third of the Swedish sample and one-quarter of the Norwegian sample responding in this manner (Table 2); these differences are statistically significant (p < 0.001) between all three countries. The proportion of RNs reporting job dissatisfaction, who indicated that they would leave the nursing profession altogether differs between countries as shown in Table 2. It should be noted however, that the total proportion of the RNs in each country sample who report an interest in leaving the nursing profession is more similar, representing 10% of the total sample from Finland, 9% of the total sample in Norway, and 7% of the total Swedish sample (data not shown). On the other hand, the proportion of the total sample in each country who reported an interest in working in a different hospital due to work dissatisfaction differed significantly (p < 0.001) between Finland, Norway and Sweden (24%, 7% and 10% respectively).

### The association between level of involvement in direct patient care and job satisfaction

In the bivariate analyses (see Table 3), the relationship between the RNs’ reported level of involvement in direct patient care and job satisfaction is most distinct in the Swedish sample. The correlation between the response alternative ‘provided most care myself’ and increased job satisfaction is not found in relation to the other response alternatives in which RNs have a more supervisory role (OR = 1.24 (1.1 ≤ CI_95%_ ≤ 1.4). This pattern is specific to the Swedish sample, and not found in either the Finnish nor Norwegian data.

**Table 3:** The association between level of involvement in direct patient care and job satisfaction

**Model**		**Finland**	**Norway**	**Sweden**
		OR (CI)*	OR (CI)*	OR (CI)*
**Bivariate analysis**				
1	“Provided most care myself” vs “supervised the care by others and provided some myself”	0.97 (0.74-1.28)	1.01 (0.87-1.17)	**1.11 (1.02-1.21)**
	“Provided most care myself” vs “provided only limited care - direct care was done by others”	0.93 (0.59-1.46)	1.13 (0.89-1.44)	**1.24 (1.10-1.40)**
	“Supervised the care by others and provided some myself” vs “provided only limited care - direct care was done by others”	0.95 (0.59-1.55)	1.12 (0.87-1.45)	1.11 (1.00-1.24)
2	Patients directly responsible for on the most recent shift (by increment of −5)	1.08 (0.97-1.19)	**1.38 (1.27-1.5)**	**1.24 (1.19-1.29)**
3	Patients required assistance with all activities of daily living (by increment of −2)	**1.07 (1.00-1.13)**	**1.29 (1.22-1.36)**	**1.31 (1.27-1.35)**
4	Patients required hourly or more frequent monitoring or treatments (by increment of −2)	**1.11 (1.03-1.19)**	**1.21 (1.14-1.29)**	**1.13 (1.10-1.16)**
Multivariate analysis**				
5	“Provided most care myself” vs “supervised the care by others and provided some myself”	0.89 (0.65-1.23)	0.86 (0.74-1.02)	**1.12 (1.02-1.23)**
	“Provided most care myself” vs “provided only limited care - direct care was done by others”	1.09 (0.65-1.82)	0.85 (0.65-1.11)	**1.17 (1.03-1.33)**
	“Supervised the care by others and provided some myself” vs “provided only limited care - direct care was done by others”	1.22 (0.72-2.06)	0.98 (0.75-1.30)	1.05 (0.93-1.17)
	Patients directly responsible for on the most recent shift (by increment of −5)	1.09 (0.96-1.24)	**1.23 (1.12-1.36)**	**1.29 (1.23-1.35)**
6	“Provided most care myself” vs “supervised the care by others and provided some myself”	0.91 (0.66-1.26)	0.86 (0.73-1.01)	1.09 (0.99-1.19)
	“Provided most care myself” vs “provided only limited care - direct care was done by others”	1.17 (0.69-1.97)	0.85 (0.66-1.11)	**1.16 (1.02-1.32)**
	“Supervised the care by others and provided some myself” vs “provided only limited care - direct care was done by others”	1.28 (0.75-2.18)	1.00 (0.75-1.31)	1.07 (0.96-1.20)
	Patients required assistance with all activities of daily living (by increment of −2)	**1.09 (1.01-1.18)**	**1.24 (1.17-1.31)**	**1.31 (1.27-1.35)**
7	“Provided most care myself” vs “supervised the care by others and provided some myself”	0.91 (0.66-1.25)	0.89 (0.76-1.04)	**1.16 (1.06-1.28)**
	“Provided most care myself” vs “provided only limited care - direct care was done by others”	1.16 (0.68-1.98)	0.92 (0.71-1.20)	**1.25 (1.10-1.42)**
	“Supervised the care by others and provided some myself” vs “provided only limited care - direct care was done by others”	1.28 (0.74-2.20)	1.04 (0.79-1.37)	1.07 (0.96-1.20)
	Patients required hourly or more frequent monitoring or treatments (by increment of −2)	**1.10 (1.01-1.19)**	**1.15 (1.08-1.23)**	**1.12 (1.09-1.15)**
8	“Provided most care myself” vs “supervised the care by others and provided some myself”	0.91 (0.68-1.21)	0.85 (0.72-1.00)	1.08 (0.98-1.19)
	“Provided most care myself” vs “provided only limited care - direct care was done by others”	0.97 (0.59-1.58)	0.85 (0.65-1.11)	**1.16 (1.02-1.32)**
	“Supervised the care by others and provided some myself” vs “provided only limited care - direct care was done by others”	1.07 (0.64-1.78)	1.00 (0.76-1.32)	1.08 (0.96-1.21)
	Patients directly responsible for on the most recent shift (by increment of −5)	0.99 (0.86-1.15)	1.03 (1.15-0.91)	**1.07 (1.02-1.13)**
	Patients required assistance with all activities of daily living (by increment of −2)	1.06 (0.97-1.16)	**1.20 (1.29-1.11)**	**1.26 (1.22-1.31)**
	Patients required hourly or more frequent monitoring or treatments (by increment of −2)	1.07 (0.99-1.16)	**1.08 (1.16-1.01)**	1.03 (1.00-1.06)

*Statistically significant values have been marked in bold.

**All multivariate analysis where also controlled for: Hospital, Total number of staff and Age, Gender and Experience of the RN.

In Sweden and Norway, job satisfaction is inversely related to the number of patients the RN reported being directly responsible for (Norway OR = 1.38 (1.27 ≤ CI_95%_ ≤ 1.5) and Sweden OR = 1.24 (1.19 ≤ CI_95%_ ≤ 1.29), as well as the number of patients with ADL (Norway OR = 1.29 (1.22 ≤ CI_95%_ ≤ 1.36) and Sweden OR =1.31 (1.27 ≤ CI_95%_ ≤ 1.35)), and surveillance (Norway OR = 1.21 (1.14 ≤ CI_95%_ ≤ 1.29) and Sweden OR = 1.13 (1.1 ≤ CI_95%_ ≤ 1.16)), needs. This indicates that an increased number of patients in general as well as increased number of patients with ADL and surveillance needs is related to higher odds of work dissatisfaction. In the Finnish sample, the number of patients with ADL (OR = 1.07 (1 ≤ CI_95%_ ≤ 1.13) and/or surveillance needs (OR = 1.11 (1.03 ≤ CI_95%_ ≤ 1.19) is inversely related to job satisfaction, although no statistically significant association is found between job satisfaction and the overall number of patients the RN was responsible for on her/his most recent shift (OR = 1.08 (0.97 ≤ CI_95%_ ≤ 1.19).

In Table 3, model 8 presents results of the multivariate analysis with all potential explanatory variables included, and controlled for total number of staff, hospital, age, sex, and length of nursing experience of respondent. The effect found in the bivariate analyses regarding the association between level of involvement in direct patient care and job satisfaction remains in the Swedish sample, but is statistically significant only in regard to differences between the most extreme response alternatives, ‘provided most care myself’ versus ‘provided only limited direct care’ (OR = 1.16 (1.02 ≤ CI_95%_ ≤ 1.32)). In the Swedish sample, the effect of increased number of patients in general (OR = 1.07 (1.02 ≤ CI_95%_ ≤ 1.13)), as well as increased number of patients with ADL needs (OR = 1.26 (1.22 ≤ CI_95%_ ≤ 1.31)), remains statistically significant related to higher odds of work dissatisfaction. In the Norwegian sample, only the effect of an increased number of patients with ADL (OR = 1.2 (1.29 ≤ CI_95%_ ≤ 1.11)) and surveillance needs (OR = 1.08 (1.16 ≤ CI_95%_ ≤ 1.01)) on job satisfaction remains statistically significant in these controlled multivariate analyses, while no statistically significant effects remain in the Finnish sample.

### The association between level of involvement in direct patient care and intention to leave current hospital

In the bivariate analyses (see Table 4), statistically significant associations were found between level of involvement in direct patient care and intention to leave in the Swedish sample, but not in the samples from Finland or Norway. The odds ratio for intention to leave was lowest among the RNs in the Swedish sample reporting a combination of direct patient care and supervisory roles (OR =0.85 (0.76 ≤ CI_95%_ ≤ 0.96)). This indicates that the relationship between level of involvement in direct patient care and intention to leave is u-shaped, with higher odds ratios for intention to leave found both among respondents reporting that they provided most care themselves, and those reporting least direct patient care.

**Table 4:** The association between level of involvement in direct patient care and intention to leave current hospital

**Model**		**Finland**	**Norway**	**Sweden**
		OR (CI)*	OR (CI)*	OR (CI)*
**Bivariate analysis**				
1	“provided most care myself” vs “supervised the care by others and provided some myself”	1.16 (0.88-1.53)	0.99 (0.83-1.18)	**1.12 (1.02-1.23)**
	“provided most care myself” vs “provided only limited care - direct care was done by others”	1.16 (0.74-1.83)	0.87 (0.66-1.15)	0.95 (0.83-1.08)
	“supervised the care by others and provided some myself” vs “provided only limited care - direct care was done by others”	1.00 (0.61-1.62)	0.87 (0.65-1.18)	**0.85 (0.76-0.96)**
2	patients directly responsible for on the most recent shift (by increment of 5)	**1.17 (1.05-1.29)**	**1.40 (1.27-1.55)**	**1.12 (1.08-1.18)**
3	patients required assistance with all activities of daily living (by increment of 2)	1.05 (0.99-1.12)	**1.26 (1.18-1.33)**	**1.21 (1.17-1.25)**
4	patients required hourly or more frequent monitoring or treatments (by increment of 2)	1.05 (0.98-1.13)	**1.20 (1.12-1.28)**	**1.13 (1.10-1.17)**
Multivariate analysis**				
5	“provided most care myself” vs “supervised the care by others and provided some myself”	1.22 (0.87-1.71)	1.05 (0.87-1.28)	**1.17 (1.06-1.30)**
	“provided most care myself” vs “provided only limited care - direct care was done by others”	0.99 (0.58-1.70)	1.12 (0.81-1.54)	1.03 (0.89-1.20)
	“supervised the care by others and provided some myself” vs “provided only limited care - direct care was done by others”	0.81 (0.47-1.41)	1.06 (0.76-1.48)	0.89 (0.79-1.02)
	patients directly responsible for on the most recent shift (by increment of 5)	1.11 (0.97-1.28)	**1.31 (1.16-1.47)**	**1.19 (1.13-1.25)**
6	“provided most care myself” vs “supervised the care by others and provided some myself”	1.21 (0.86-1.69)	1.04 (0.85-1.26)	**1.21 (1.09-1.34)**
	“provided most care myself” vs “provided only limited care - direct care was done by others”	0.94 (0.54-1.62)	1.08 (0.79-1.48)	1.04 (0.90-1.20)
	“supervised the care by others and provided some myself” vs “provided only limited care - direct care was done by others”	0.78 (0.44-1.36)	1.04 (0.75-1.45)	**0.87 (0.77-0.99)**
	patients required assistance with all activities of daily living (by increment of 2)	1.08 (0.99-1.18)	**1.23 (1.15-1.32)**	**1.22 (1.17-1.26)**
7	“provided most care myself” vs “supervised the care by others and provided some myself”	1.21 (0.86-1.69)	1.02 (0.84-1.24)	1.17 (1.06-1.29)
	“provided most care myself” vs “provided only limited care - direct care was done by others”	0.93 (0.53-1.62)	1.02 (0.75-1.40)	0.98 (0.85-1.13)
	“supervised the care by others and provided some myself” vs “provided only limited care - direct care was done by others”	0.77 (0.43-1.36)	1.00 (0.72-1.4.0)	0.87 (0.76-0.99)
	patients required hourly or more frequent monitoring or treatments (by increment of 2)	1.03 (0.94-1.12)	1.18 (1.10-1.28)	1.13 (1.10-1.17)
8	“provided most care myself” vs “supervised the care by othersand provided some myself”	1.24 (0.88-1.74)	1.07 (0.88-1.30)	**1.21 (1.09-1.34)**
	“provided most care myself” vs “provided only limited care - direct care was done by others”	0.92 (0.52-1.61)	1.12 (0.81-1.54)	1.02 (0.88-1.18)
	“supervised the care by others and provided some myself” vs“provided only limited care - direct care was done by others”	0.74 (0.41-1.31)	1.05 (0.75-1.47)	**0.86 (0.76-0.98)**
	patients directly responsible for on the most recent shift (by increment of 5)	1.06 (0.88-1.26)	1.09 (0.95-1.26)	1.01 (0.95-1.07)
	patients required assistance with all activities of daily living (by increment of 2)	1.07 (0.96-1.19)	**1.16 (1.06-1.26)**	**1.18 (1.13-1.23)**
	patients required hourly or more frequent monitoring or treatments (by increment of 2)	0.99 (0.90-1.09)	**1.11 (1.02-1.20)**	**1.08 (1.04-1.11)**

*Statistically significant values have been marked in bold.

**All multivariate analysis where also controlled for: Hospital, Total number of staff and Age, Gender and Experience of the RN.

As was the case for job satisfaction, in Sweden and Norway intention to leave one’s current hospital was found to be associated with increased number of patients the RN reported being directly responsible for (Sweden OR = 1.12 (1.08 ≤ CI_95%_ ≤ 1.18), Norway OR = 1.4 (1.27 ≤ CI_95%_ ≤ 1.55)), as well as increased number of patients with ADL (Sweden OR = 1.21 (1.17 ≤ CI_95%_ ≤ 1.25), Norway OR = 1.26 (1.18 ≤ CI_95%_ ≤ 1.33)) and surveillance needs (Sweden OR = 1.13 (1.1 ≤ CI_95%_ ≤ 1.17), Norway OR = 1.2 (1.12 ≤ CI_95%_ ≤ 1.28)). In the Finnish sample, the total number of patients the RN is responsible for is directly related to intention to leave (OR = 1.17 (1.05 ≤ CI_95%_ ≤ 1.29)).

In the multivariate analyses (see Table 4), the general patterns noted in the bivariate analyses between the RNs’ reported level of involvement in direct patient care and intention to leave remain. In Table 4, model 8 presents results of the multivariate analysis with all potential explanatory variables related to intention to leave included, and controlled for total number of staff, hospital, age, sex, and length of nursing experience of respondent. In both Norway and Sweden, the associations between the number of patients with ADL (Norway OR = 1.16 (1.06 ≤ CI_95%_ ≤ 1.26), Sweden OR = 1.18 (1.13 ≤ CI_95%_ ≤ 1.23)) and surveillance needs (Norway OR = 1.11 (1.02 ≤ CI_95%_ ≤ 1.2), Sweden OR = 1.08 (1.04 ≤ CI_95%_ ≤ 1.11)) remains statistically significant when controlled for the above-named variables. In the Finnish sample, no significant associations between these variables are found in the multivariate analyses.

## Discussion

Despite the many similarities otherwise found in these health care systems, in this data set from three Nordic countries we find notable differences in the role of RNs in patient care, the mean number of patients the RNs are directly responsible for, and in the number of patients needing ADL assistance and surveillance by the RNs. Regardless of these differences, most RNs report being generally satisfied with their work and would recommend their work place to colleagues, with the level of very dissatisfied RNs only 3-4% in all three countries. However, close to half of the Finnish sample reports that they would leave their current hospital- within the next year due to work dissatisfaction if possible, with the comparable responses in the other two Nordic countries significantly lower. Patient workload is to some degree associated with both job satisfaction and intention to leave in all three countries, such that the greater the patient workload, the less job satisfaction and greater intention to leave. Even when controlling for a series of other relevant variables, some associations remain statistically significant in the data from Norway and Sweden, although this is not the case for the Finnish data.

However, it should be noted that the Finnish sample is also the smallest, and may thus have insufficient power in the more complex regression models. The differences in recruitment strategies and data collection approaches should also be considered when interpreting these results. For example, whereas the Swedish data is based on a population drawn from the over 80% of unionized RNs, the samples in Finland and Norway are selected by hospital and unit. No significant differences were found in the composition of the respondents versus non-respondents in the Swedish sample, and we see no reason to assume that this would differ among countries. Despite these differences in recruitment in these countries, this data set is based on the same, stringently translated and validated instruments in large samples of RNs, with data collected at the same points in time thus providing a unique opportunity to explore some questions previously not investigated.

We found notable differences in intention to leave their current hospital- among RN respondents in these three countries. Among the 12 European RN4CAST countries the respondents from Finland, along with those from Greece, have the highest proportion of RNs reporting intention to leave. Norway, on the other hand, is among the three countries with less than 30% of respondents reporting intention to leave in the RN4CAST material [13]. In contrast, the proportion of the total sample in each of the three Nordic countries reporting interest in leaving the nursing profession altogether is similar-- under 10%. This figure is much lower than that found by Flinkman et al. [14] in their survey of 147 young RNs in six hospital districts in Finland. It does not however differ as much from results of the NEXT study, where the percentage of RNs frequently considering leaving the profession was about 14% in Finland and 12% in Norway^a^ ([15], p19). What might constitute a normal level of attrition in any profession should also be considered. It is possible that most effort should be focused on retaining those RNs who are otherwise satisfied with their choice of profession, but have grievances at a particular workplace, thus maintaining a focus on improving those factors which can be changed in work environments. This is in line with Sjögren et al’s Swedish study [16], finding that the primary reasons given by nursing staff (including RNs and nursing assistants) for considering leaving the profession were related to working and employment conditions.

Perhaps the most notable results of this study are the clear differences in the role of the RN in patient care in these three Nordic countries, where RNs from the Swedish sample report having a supervisory role to a greater extent than do RNs in Finland and Norway. The finding that RNs in Sweden are more satisfied in roles that allow for more direct patient care suggests that it may be feasible to recruit RNs into positions in greater proximity to patients. The lack of relationship found in this regard in Finland and Norway, may well reflect differences in organization, with a notably higher proportion of RN’s already working in direct patient care.

The u-formed relationship between patient care role and intention to leave found in the Swedish data, is particularly intriguing. One possible interpretation is that this may in part reflect position in hospital hierarchies that lack clear career ladders for RNs. Sweden, for example, has no national or regional formalized system for clinical career ladders, neither for salary nor for continued education, although individual workplaces may have their own policies. It may well be that those RNs responsible for most direct patient care are those with lowest status. Those with most supervisory functions may find themselves too distant from aspects of nursing they originally found satisfying, or may have reached a final post in an existing career hierarchy.

There are however, a number of issues to consider in interpreting these results. One possibility is if the items, however stringent the translation process, may be understood differently in different countries, although we have no support for this interpretation. Another issue is that of auxiliary nursing staff, their qualifications and their level of involvement in direct patient care. Despite apparent similarities in qualifications and terminology, the differences in how auxiliary nursing staff functions in different countries may well be parallel to those differences found among RNs in these Nordic countries. This would be expected to affect not only patient care, but also nursing organization and management, and would thus be of interest for further study. An additional potential bias might be differences in strategies for data collection in the three counties. In Sweden and Finland, participants were not recruited directly at their workplace, although this was the case in Norway. However while other RN4CAST countries recruited via hospital management, this was not the case in these Nordic countries; on the contrary both Sweden and Norway collaborated with the national organizations for nurses. It is unclear if and what affect this may have on participation or responses as it was made clear that independent researchers received the data in all three countries.

Although the patterns in response concerning level of involvement in direct patient care are similar in Finland and Norway, the patient: nurse ratio is higher in Finland than in the other countries. This remains the case even when the patient: total nursing staff ratio is considered. In addition, the RNs in the Finnish sample report caring for more patients with ADL and surveillance needs than do the RNs in the other two countries. This may be in part related to both the short lengths of stay for surgical patients in particular ([17] page 278: Finland 2.8 days vs. 4.3 in Norway and 4.1 in Sweden) and the lesser number of hospital beds, particularly for medicine, in acute care hospitals in Finland (102 per 100 000 persons in Finland, compared to 136 and 150 in Norway and Sweden respectively [17] p 277); these factors may all contribute not only to evaluations of greater cost efficiency in Finnish hospitals than those in Norway and Sweden [5,18], but also to the greater patient workload and the higher levels of dissatisfaction reported by the RNs in Finland. Research suggests that variation in human resources utilization may be part of the explanation for differences in cost efficiency among countries [5,18], but the potential costs of overburdened or dissatisfied RNs have not been considered to date in such evaluations.

It may not be surprising that number of patients per RN or the intensity of care required may contribute to dissatisfaction and intention to leave, but the fact that this effect remains in the large Swedish data sample even after controlling for total number of nursing staff, including non-RNs, might imply that other, less apparent factors may be of importance. While it is well recognized that other aspects of the working environment are relevant (see for example [19-22]), the size of a unit might influence job satisfaction. A question that might be asked in future research is if the number of people—both patients and staff—that an RN interacts with on a given shift is of importance, rather than the patient: nurse ratio or intensity of surveillance and ADL needs only. We lack data to investigate this further in the present material.

In summary, this article presents the first comprehensive analysis of issues related to organization of nursing care in these Nordic countries, with large data sets systematically collected to allow comparisons. Other comparative studies have not included nursing issues, in part because stringent and comparable data has been lacking to date (see for example [5]. While it is possible that the levels of intention to leave their current hospital reported here may not be fully actualized in practice, it has previously been found that intention to leave is a viable predictor of actual behavior [15]. If so, the data from this study represent a cause for concern for the future. On the other hand, these results also suggest a number of factors which may be susceptible to constructive change. These include attention paid to patient mix and workload as well as the role of the nurse in patient care. The latter factor has not been the subject of much attention to date, but appears to be of importance. If RNs are to be enticed to remain in their organizations and in the countries in which they are educated, more attention needs to be paid to remedying those factors found to be related to dissatisfaction and intention to leave. Without adequate numbers of RNs to sustain health care organizations, high quality patient care is jeopardized, despite other resources and organizational efforts.

## Conclusions

Despite the many similarities otherwise found in the Nordic health care systems, we find notable differences in the level of involvement of RNs in direct patient care, the mean number of patients the RNs are directly responsible for, and in the number of patients needing ADL assistance and surveillance by RNs. Patient workload is associated with job satisfaction and intention to leave— the greater the patient workload, the less job satisfaction and greater intention to leave. This is the case even when number of total nursing staff is considered. In the largest national sample, with most variation in degree of direct patient care, RNs’ level of involvement in direct patient care was found to be related to their job satisfaction. RNs working with direct patient care were more likely to be satisfied than were RNs in supervisory roles with little direct patient care. One implication of these findings is that when making efforts to improve staff retention, policymakers and management should not only consider workload and patient mix, but also consider the role of the RN in patient care.

## Endnote

^a^There is no published data from Sweden for this question in the NEXT study.

## Competing interest

The authors declare that they have no competing interest.

## Authors’ contributions

All authors (RL, LSA, SR, AE, VJ, JH, ISS, CT MWT and CaT) were actively involved in the design of the study presented here. CaT, RL, and SR were responsible for data collection in Sweden. AE,VJ and JK were responsible for the data collection in Finland and ISS, CT and MWT were responsible for the data collection in Norway. AE,VJ and JK actively participated in writing the portions of the article based on Finish health care and data, and ISS, CT and MWT for corresponding sections for Norway. RL led, with CaT, writing of the first and successive drafts of the paper. SR carried out statistical analyses and contributed to the writing of the manuscript. All authors contributed to interpretation of the results, commenting and revising the text. All authors approved of the final version.

## Pre-publication history

The pre-publication history for this paper can be accessed here:

http://www.biomedcentral.com/1472-6955/13/27/prepub
